# Changes in lower limb rotation after soft tissue surgery in spastic diplegia: 3-dimensional gait analysis in 28 children

**DOI:** 10.3109/17453671003587135

**Published:** 2010-04-06

**Authors:** Bjørn Lofterød, Terje Terjesen

**Affiliations:** ^1^Section for Child NeurologyNorway; ^2^Department of Orthopaedics, Rikshospitalet University Hospital and Faculty of Medicine, University of Oslo, OsloNorway

## Abstract

**Background and purpose** Rotational osteotomies are usually necessary to correct pronounced rotational deformities in ambulant children with cerebral palsy. The effects of soft tissue surgery on such deformities are unclear. In this retrospective study, we determined whether multilevel soft tissue surgery, performed to correct deformities in the sagittal plane, would also have an effect on rotational parameters.

**Patients and methods** We examined 28 ambulant children with spastic diplegia with an average age of 12 (7–19) years. They underwent multilevel soft tissue surgery (with 6 surgical procedures per child on average). 3-dimensional gait analysis was performed preoperatively and at an average follow-up of 1–2 years. The indications for surgery were abnormalities in the sagittal plane. Gait analysis data from healthy children were used in defining normal ranges of kinematic variables. For assessment of changes in the transverse plane, the angles of foot progression, hip rotation, and pelvic rotation were studied.

**Results** The transverse plane kinematic results showed no statistically significant postoperative changes when the preoperative parameters were within the normal range (within 2 SD of the mean of the normal material). In limbs where the preoperative values were abnormal (more than 2 SD above the normal mean), there was a mean reduction in internal foot progression of 12° (p = 0.01) and a mean reduction in external pelvic rotation of 6° (p = 0.02). The effect was more pronounced in children under 12 years of age. Internal hip rotation was not significantly reduced.

**Interpretation** When the preoperative rotational parameters were abnormal, multilevel soft tissue surgery resulted in improved transverse plane kinematics. This could be of importance in preoperative decision making, especially when there is doubt as to whether to include rotational osteotomies in multilevel operations in younger children.

## Introduction

The goal of surgery on the lower extremities in ambulant children with spastic cerebral palsy is to improve gait function, which often includes normalization of foot progression and correction of lever-arm dysfunction. Increased internal or external foot progression is often caused by malrotations of proximal bones and joints, and rotational osteotomies may be necessary to correct the abnormality. Multilevel surgery of the lower extremity means procedures in which all muscle contractures and lever-arm dysfunctions are corrected during a single operation (Gage and Novacheck 2001). This type of surgery can be very comprehensive and the postoperative rehabilitation is demanding for the children and their parents, especially if bony procedures have been involved.

Most former studies on rotational deformities have focused on the effects of rotational osteotomies (Stefko et al. 1998, Ounpuu et al. 2002). Only a few studies have assessed the results of soft tissue surgery. Moreover, previous studies of the effect on hip rotation and pelvic rotation have found divergent results (Steinwender et al. 2000, Kay et al. 2004, Lovejoy et al. 2007).

We evaluated whether multilevel soft tissue surgery, performed to correct deformities in the sagittal plane, would also have an effect on rotational deformities.

## Patients and methods

The study included 28 ambulant children (16 boys) with spastic diplegia who had undergone soft tissue surgery on their lower limbs between January 2002 and December 2006. The average age at the time of operation was 12 (7–19) years. Children who had concomitant rotational osteotomy of the femur or tibia, or bony surgery of the foot (such as subtalar arthrodesis) were not included. Patients with isolated calf muscle lengthening were not included because our experience with this procedure has been published previously (Lofterød and Terjesen 2008a). The study was approved by the Data Protection Officer according to paragraph 26 of the Norwegian Health Personnel Act.

The gait function was classified according to the Gross Motor Function Classification System (GMFCS) (Palisano et al. 1997). 6 children were classified at level I, 14 at level II, and 8 at level III. 16 children (31 lower limbs) had previously undergone orthopedic surgery (Table 1). None of the children had had botulinum toxin injections within 6 months before the preoperative gait analysis. 1 child had continuous intrathecal baclofen therapy and had unchanged dosage pre-and postoperatively.

**Table 1.** Type and number of previous surgical procedures and procedures evaluated in the present study

Type of procedure	Previous procedures	Present procedures
Calf muscle lengthening	25	18
Hamstring tenotomy	10	49
Rectus femoris transfer	–	44
Adductor release	10	17
Psoas tenotomy	4	38
Femoral osteotomy	6	–
Tibial osteotomy	1	–
Foot operation	8	–
Total	64	166

All the children underwent pre-and postoperative clinical examination and gait analysis. A 6-camera Vicon System (612; Oxford Metrics, Oxford, UK) and 2 AMTI force plates (Advanced Mechanical Technology Inc., Watertown, MA) were used to collect motion analysis data. The average time between preoperative gait analysis and surgery was 8 (2–14) months and the mean time between operation and postoperative gait analysis was 14 (12–25) months.

To compare pre-and postoperative kinematic data obtained by gait analysis, a representative trial from each subject was selected for analysis as described by Ounpuu et al. (2002). If all trials showed similar patterns based on visual evaluation of the plots, the initial trial was selected. If 2 of 3 trials were similar, the first trial (of the 2) was selected.

27 children were operated in our hospital by 3 different surgeons and 1 child was operated in another hospital by a cooperating surgeon using the same methods. 166 surgical procedures were performed (Table 1). The most frequent procedures were medial hamstring tenotomies (49) and rectus femoris transfer to sartorius (44). 53 limbs were operated and 25 of the 28 children had bilateral procedures. There was an average of 6 surgical procedures per child and 3 procedures per limb.

As a help in defining the indications for surgery, gait data from a normal material was used (Table 2). This consisted of 24 healthy children (13 boys) with a mean age of 10 (5–15) years. They were examined in our gait laboratory. The children were recruited from parents working at the hospital. The range of normal variation was defined as mean ± 2 SD. Preoperative kinematic parameters in the sagittal plane outside this range were used as indications for surgery in children with diplegia. Maximum ankle dorsiflexion in stance below 5° indicated calf muscle lengthening, hamstring tenotomy was discussed when minimum knee flexion in stance was above 10°, and flexion contracture of the hip led to psoas tenotomy when minimum hip flexion in stance was above 0°. The indication for rectus femoris transfer was based on 3 parameters: maximum knee flexion in swing (less than 50°), timing of knee flexion in swing (more than 5% delayed), and total knee range of motion (less than 45°). At least 2 of these 3 parameters should be abnormal.

**Table 2.** Pre- and postoperative transverse plane kinematics when the preoperative kinematic parameters were between 1 SD and 2 SD above the mean value of the normal material. Results are mean values in degrees (negative value is external rotation)

Rotational parameter		Children with diplegia				Normal material	
	n	Pre-operatively	Post-operatively	Mean difference (95% CI)	p-value	Mean (SD)	Mean + 1 SD
Foot progression	4	4	2	2 (-10 to 13)	0.7	-5 (7)	2
Hip rotation	9	11	13	-2 (-9 to 5)	0.6	1 (7)	8
Pelvic rotation	7	-6	-2	4 (-9 to 1)	0.07	0 (4)	-4
n: number of children; CI: confidence interval.							

The following pre-and postoperative kinematic parameters in the transverse plane were studied: mean foot progression in stance, mean hip rotation in stance, and mean pelvic rotation in stance. Because the statistics can be unreliable when both limbs are included (Bryant et al. 2006), only one operated limb of each child was included in the statistical analysis. The limb with the highest preoperative value was chosen for the analysis of internal foot progression and the limb with the highest preoperative internal hip rotation was chosen for the analysis of hip rotation. Since pelvis is one segment, the side with external rotation (retraction) was selected for analysis.

### Statistics

The significance level (p-value) was set at 0.05. The paired samples t-test was used to compare the pre- and postoperative kinematic parameters. Pearson's coefficient of correlation was used to assess the relationship between the rotational variables.

## Results

2 of the 28 children had improved GMFCS level. 1 child improved from level III to level II, and another child improved from level II to level I. None of the children had deterioration of GMFCS level. The multilevel soft tissue procedures effectively corrected the deformities in the sagittal plane. In the 18 feet in which calf muscle lengthening had been performed, the mean maximum ankle dorsiflexion in the stance phase increased from –1° to 12° (p = 0.005). Mean minimum knee flexion in stance decreased from 31° to 12° (p < 0.001) in the 49 limbs where hamstring lengthening was performed, and mean minimum hip flexion in stance decreased from 13° to 6° (p < 0.001) in the 38 limbs in which psoas lengthening had been done.

Although the mean values of the preoperative rotational kinematic parameters were within the normal range, many children had moderately increased (between 1 SD and 2 SD above the mean of the normal material) or abnormally high preoperative values (above mean plus 2 SD of the normal material). In children with moderately increased preoperative rotational parameters, there were no statistically significant differences postoperatively (Table 2). In children with abnormally high preoperative internal foot progression and pelvic retraction, there was a statistically significant improvement in kinematics with a mean reduction of 11° and 7°, respectively. Internal hip rotation decreased 3° but this difference was not statistically significant (Table 3).

**Table 3.** Pre- and postoperative transverse plane kinematics when the preoperative kinematic parameters were above 2 SD of the mean value of the normal material. Results are mean values in degrees (negative value is external rotation)

Rotational parameter		Children with diplegia				Normal material	
	n	Pre-operatively	Post-operatively	Mean difference (95% CI)	p-value	Mean (SD)	Mean + 2 SD
Foot progression	14	19	8	11 (3 to 21)	0.01	-5 (7)	9
Hip rotation	14	21	18	3 (-1 to 7)	0.1	1 (7)	15
Pelvic rotation	12	-13	-6	7 (2 to11)	0.02	0 (4)	-8
n: number of children; CI: confidence interval.							

In order to evaluate whether age at surgery had an influence on the results, the children were divided into 2 groups: under 12.1 years (16 children) and over 12.1 years (12 children). Only children with preoperative transverse plane kinematics of more than +1 SD above the mean of the normal material were assessed. Statistically significant improvements in pelvic retraction occurred in both age groups, whereas internal foot progression was significantly improved only in children under 12.1 years of age. The differences in hip rotation were not significant in any of the age groups (Table 4).

**Table 4.** Pre- and postoperative transverse plane kinematics according to the age of the children (under 12.1 years and over 12.1 years). Only children with preoperative kinematic parameters above 1 SD of the mean value of the normal material have been included. Results are mean values in degrees (negative value is external rotation)

Rotational parameter		Children under 12.1 years					Children older than 12.1 years			
	n	Pre-operatively	Post-operatively	Difference (95% CI)	p-value	n	Pre-operatively	Post-operatively	Difference (95% CI)	p-value
Foot progression	8	14	-3	(2 to 31)	0.03	10	18	14	(-2 to 10)	0.2
Hip rotation	12	16	14	(-4 to 7)	0.5	11	18	18	(-5 to 7)	0.8
Pelvic rotation	10	-10	-3	(-13 to -2)	0.02	9	-10	-7	(-6 to 0)	0.04
n: number of children; Pre: preoperatively; Post, postoperatively; CI: confidence interval.										

Preoperatively, there was a correlation between pelvic rotation and hip rotation (r = –0.41; p = 0.03). Postoperatively, no statistically significant correlations between the rotational parameters were found. There was a correlation between the pre- to postoperative changes in foot progression and pelvic rotation (r = 0.55; p = 0.002).

## Discussion

The multilevel soft tissue procedures were performed because of abnormalities in the sagittal plane. The results confirmed improved sagittal plane kinematics, with better extension of the hips and knees and better dorsiflexion of the ankle in the stance phase. This has been shown in previous studies; it is mentioned here only to indicate that the surgery had caused the main effects required. The present study, however, focused on the side effects of soft tissue surgery on the most common rotational parameters, which has rarely been addressed in previous studies.

Preoperatively, several children had abnormal kinematic variables in the transverse plane, but rotational osteotomy was not performed. Usually, we consider femoral osteotomy when internal femoral rotation in the stance phase is above 20° (Lofterød and Terjesen 2008b). 4 children (6 limbs) had increased femoral rotation within the range of 21–37°. Femoral derotation was discussed, but for various reasons it was not performed, which included previous bilateral femoral osteotomies in 1 child and increased pelvic retraction and normal foot progression in another. Also, parents or doctors may prefer a smaller operation with an easier rehabilitation period, which is sometimes more important in preoperative decision making than abnormal angles at gait analysis.

In limbs with abnormally high preoperative transverse plane values, multilevel soft tissue surgery resulted in reduced pelvic retraction and reduced internal foot progression. Abnormal pelvic retraction is quite common in spastic cerebral palsy; a frequency of 0.5 in hemiplegia and 0.3 in diplegia was reported by O'Sullivan et al. (2007). A somewhat higher frequency (12 of 28 children) was found in our study. O'Sullivan et al. (2007) suggested that pelvic retraction is multifactorial in origin and often associated with increased internal hip rotation in spastic diplegia. Such a correlation was also present in our children.

Kay et al. (2004) reported a mean decrease of 3° in pelvic retraction after soft tissue surgery. Steinwender et al. (2000) found no effect on pelvic rotation, but they included the pelvis on both sides, which is hardly adequate because external rotation of one hemipelvis cancels internal rotation of the other side. In accordance with Kay et al. (2004), we included only the side with more external pelvic rotation. We found no effect on pelvic retraction in children with preoperative external rotation within the normal range, whereas children with preoperative retraction more than mean + 2 SD of the control group had a mean improvement of 7° after surgery. This relationship between the degree of preoperative pelvic rotation and surgical effect does not appear to have been studied in previous reports.

20 of the 53 limbs had preoperative foot progression within mean ± 1 SD of the control group whereas 27 limbs had more in-toeing than this level, and 6 limbs had more out-toeing. Thus in-toeing was considerably more frequent than out-toeing. Rethlefsen et al. (2006) found that in-toeing was most often associated with increased internal hip rotation. We found no statistically significant preoperative correlations between foot progression and either hip rotation or pelvic rotation.

Several reports have attributed internal hip rotation to spastic medial hamstrings or adductors (Banks and Green 1960, Majestro and Frost 1971, Chong et al. 1978, Steinwender et al. 2000). More recent research has, however, revealed that the medial hamstrings, adductor longus, brevis, and gracilis have negligible internal rotation moment arms in children with cerebral palsy who walk with a crouched, internally rotated gait (Arnold et al. 2000). These muscles have therefore been considered unlikely to be important contributors to excessive internal rotation of the hip. In our study, the effect of soft tissue surgery on internal hip rotation tended to increase somewhat with increasing preoperative rotation. However, the differences were not statistically significant, indicating no reliable effect, which is in keeping with the work of Kay et al. (2004). These results seem to be in sharp contrast to those of Steinwender et al. (2000), who reported a marked improvement of no less than 12° after soft tissue surgery and attributed this positive effect to hamstring lengthening. If hamstring lengthening was so important, a similar effect would be expected in our study where hamstring lengthening was performed in almost all the operated limbs. Lovejoy et al. (2007) reported a statistically significant decrease in internal hip rotation of almost 5° after soft tissue surgery. They felt that this effect was caused by lengthening of the medial and lateral hamstrings, which was performed in all the limbs. There was no increased effect in limbs with increased internal hip rotation preoperatively.

Kay et al. (2004) reported a mean decrease in internal foot progression of 5° but did not mention whether the decrease was larger in limbs with abnormally high preoperative foot progression. We found that the difference between pre-and postoperative foot progression increased with increasing preoperative values. In limbs with abnormally high preoperative internal foot progression the mean improvement was 11°, which is quite satisfactory from a clinical point of view. The effect was, however, less than that reported after derotational femoral osteotomies, where a mean improvement of 16–21° occurred (Ounpuu et al. 2002, Kay el al. 2003).

The effect of soft tissue surgery on foot progression and pelvic retraction was more pronounced in children under 12 years of age at surgery. This indicates that improvement in rotational parameters is more likely to occur in younger children, probably because they have a somewhat more immature gait pattern. The influence of age appears not to have been examined in previous studies.

The mean time between operation and postoperative gait analysis was 14 months. The results must therefore be considered as short-term outcome. As a routine in our laboratory, all the children have a gait analysis 5 years after the operation. By the closure of this study, 4 of the 28 children had undergone the 5-year gait analysis and none of them were in need of bone surgery for corrections in the transverse plane.

We did not analyze knee rotation in this study. There are concerns about the ability of standard motion analysis equipment and methods to measure this angle accurately (Scwartz 2004). To increase the reliability of hip rotation, we used the technique described by Baker et al. (1999). The degree of reliability depends much on the routines in the laboratory. In our lab, the same set of personnel (child neurologist, orthotist, physiotherapist, and orthopedic surgeon) performed the clinical examination, executed the analysis, and made team conclusions. In this way, the risk of interobserver variation was reduced compared to when several separate observers are involved (Skaggs et al. 2000). Teamwork enabled us to follow the same standards and to discuss and correct any deviation that could occur. In this study, the first author selected all the trials and thereby limited any inconsistency in selecting a representative trial.

When to perform a bony procedure is unclear. We agree with others (Arnold et al. 2000, Rethlefsen et al. 2006, Lovejoy et al. 2007) that soft tissue surgery should not be recommended to correct pronounced internal rotation, as this is almost universally associated with bony deformity rather than with spastic muscles alone and need rotational osteotomy for adequate correction. Even so, in more moderate rotational deformities and when there is doubt as to whether to perform a large operation including bony surgery, an improvement in rotational deformities can be expected as a side effect following soft tissue procedures aimed at correction of deformities in the sagittal plane.
